# Phytochemical Characteristics and Antimicrobial Activity of Australian Grown Garlic (*Allium Sativum* L.) Cultivars

**DOI:** 10.3390/foods8090358

**Published:** 2019-08-23

**Authors:** Anh Dao Thi Phan, Gabriele Netzel, Panhchapor Chhim, Michael E. Netzel, Yasmina Sultanbawa

**Affiliations:** ARC Training Centre for Uniquely Australian Foods, Queensland Alliance for Agriculture and Food Innovation, The University of Queensland, Brisbane 4072, Queensland, Australia

**Keywords:** Australian grown garlic, *Allium sativum* L., polyphenols, organosulfur compounds, antioxidant capacity, antimicrobial activity

## Abstract

This study systematically evaluated the main bioactive compounds and associated biological properties of two Australian grown garlic cultivars and commercial non-Australian grown garlic (for comparison purposes only). Additionally, the distribution of bioactive compounds in garlic skin and clove samples was determined to obtain a better understanding of the potential biological functionality of the different garlic parts. The identification and quantification of bioactive compounds was performed by ultra-high performance liquid chromatography with mass spectrometry and photodiode array detection (UHPLC-PDA-MS). A principal component analysis was applied to assess the correlation between the determined bioactive compounds and antioxidant capacity as well as antimicrobial activity. The content of phenolic compounds (free and bound forms) in the garlic skin samples was significantly (*p* < 0.05) higher than that of the garlic cloves, and was also higher (*p* < 0.05) in the Australian grown cultivars compared to the commercial non-Australian grown garlic. Anthocyanins were found in the skin samples of the Australian grown garlic cultivars. The organosulfur compounds were higher (*p* < 0.05) in the cloves compared to the skin samples and higher (*p* < 0.05) in the Australian grown cultivars compared to the studied commercial sample. As the richer source of bioactive compounds, the Australian grown garlic cultivars exhibited a significantly (*p* < 0.05) higher antioxidant capacity and stronger (*p* < 0.05) antimicrobial activity than the commercial non-Australian grown garlic. The potential of garlic cultivars rich in bioactive compounds for domestic and industrial applications, e.g., condiment and natural food preservative, should be explored further.

## 1. Introduction

Garlic (*Allium sativum* L.) has been known as “aroma” vegetable, which is widely used as a food ingredient in many countries and different cultures as a result of its characteristic flavor and potential health benefits. Many studies have shown evidence of a significant reduction of the risk of developing chronic diseases (e.g., cardiovascular, cancer, obesity, diabetes, high blood pressure, platelet aggregation, cholesterol lowering) associated with garlic consumption [1,2,3,4,5]. Together with therapeutic functions, garlic possesses additional biological activities such as antibacterial, antifungal, and antioxidant properties [6,7,8], resulting in garlic being one of the most important vegetables worldwide [9]. 

It has been suggested that the biological and health properties of garlic are derived from its polyphenols and organosulfur compounds. Garlic possesses γ-glutamyl-S-alk(en)yl-L-cysteines and S-alk(en)yl-L-cysteine sulfoxides, particularly L-alliin as the major sulfur-containing compound in intact garlic [2]. Under different physical treatments (e.g., cutting, crushing, or chewing), the enzyme alliinase, released from the vacuole, lyses the S-alk(en)yl-L-cysteine sulfoxides to liberate the majority of the characteristic aroma thiosulfinate compounds such as allicin, diallyl sulfide, and diallyl disulfides [10,11]. These volatile compounds are extremely unstable and rapidly decomposed to form other sulfur-containing compounds, which might not be the genuinely active compounds of garlic [12]. 

In addition to organosulfur compounds, garlic contains a diverse range of phenolic compounds such as phenolic acids [13,14,15] and anthocyanins [16,17]. Whilst organosulfur compounds are extremely unstable and susceptible to further transformation, recent attention has been placed on polyphenols due to their potential role in health-related benefits for humans [1]. Apart from its phenolic and organosulfur compounds, garlic is also rich in vitamins and minerals [18]. However, it should be noted that the content of these bioactive compounds can vary depending on the genotype, agronomic conditions, environmental factors, maturity, and post-harvest conditions [18,19,20]. It has been reported that the total phenolic content decreases with the increase in organosulfur compounds and terpenoid substances in mature garlic bulbs [15].

The available information on the phytochemical composition, tissue distribution (clove versus skin) and bioactive properties of Australian grown garlic is very limited. Therefore, the aim of the present study was (*i*) to generate crucial nutritional data, including the proximate composition, minerals, heavy metals, polyphenols, and organosulfur compounds of Australian grown garlic, (*ii*) to determine the distribution of polyphenols and organosulfur compounds within garlic (cloves versus skin), *(iii)* to evaluate the antioxidant and antimicrobial activities, and (*iv*) to prove the potential correlations between observed biological activities and determined bioactive compounds using principal component analysis (PCA).

## 2. Materials and Methods 

### 2.1. Materials

Fresh Australian grown garlic (Cultivars X and Y) were supplied from field samples grown in St. George, Queensland, Australia. The cultivars X and Y were breeding lines that are being trialed in Queensland and not available for commercial production yet. Commercial non-Australian grown garlic (product of China) was purchased from a local supermarket in Brisbane, Queensland, Australia, and was included for comparison. Fresh garlic samples were separated into cloves and skin and then freeze-dried at −50 °C for 48 hours (CSK Climatek, Darra, Queensland, Australia). The lyophilized materials were then ground to a very fine powder using a milling machine (Foss Cyclotec Sample Mill, Mulgrave, Victoria, Australia), and stored in airtight containers at −35 °C for further analysis.

Phenolic standard compounds and L-alliin were HPLC grade and purchased from Sigma-Aldrich (Castle Hill, New South Wales, Australia). 

The following microbial cultures, included Gram-positive bacteria (*Bacillus cereus* ATCC 10876, *Listeria monocytogenes* ATCC 19111; American Type Culture Collection, In Vitro Technologies Pty Ltd., Noble Park Victoria, Australia, and *Staphylococcus aureus* NCTC 6571; National Collection of Type Cultures, Health Protection Agency Centre for Infection, London, UK), Gram-negative bacteria (*Pseudomonas aeruginosa* ATCC 10145, *Escherichia coli* NCTC 9001), and yeasts (*Candida albicans* ATCC 10231 and *Rhodotorula mucilaginosa* from the Culture Collection of the Centre for Nutrition and Food Sciences, The University of Queensland, Queensland, Australia) were used for the antimicrobial test.

Plate count agar medium (PCA) (Oxoid, CM0325, Thermo Fisher Scientific Pty Ltd., Scoresby, Victoria, Australia) and potato dextrose agar medium (PDA) (Oxoid, CM0139 Thermo Fisher Scientific Pty Ltd.) were used to determine the antibacterial and antifungicidal activity, respectively. 

### 2.2. Proximate Analysis 

Proximate analysis were performed on the freeze-dried powder of the edible garlic cloves at Symbio Alliance Laboratories (Eight Mile Plains, Queensland, Australia), which is a National Association of Testing Authorities (NATA) accredited laboratory that complies with International Organization for Standardization/the International Electrotechnical Commission (ISO/IEC) 17025:2005. The analysis was done according to the NATA approved in-house methods or the Association of Official Analytical Chemists (AOAC) methods as follows: Protein by AOAC method 990.03 (AOAC, 1997); fat by AOAC method 991.36 (AOAC, 1999); saturated, monounsaturated, polyunsaturated, and trans fatty acids by gas chromatography with flame-ionization (in-house method CFH068.2); moisture by AOAC method 934.01 (AOAC, 1990); ash by AOAC method 923.03 (AOAC, 2000); minerals and heavy metals by inductively coupled plasma mass spectrometry method (ICP_MS); total sugar, total dietary fiber, and available carbohydrates by high performance liquid chromatography with refractive index detection (in-house methods CFH001.1, CF057, and CF029.1, respectively); energy based on calculation from proximate data (in-house method CF030.1); crude fiber by AOAC method 962.09 (AOAC, 1990); dry matter by in-house method CF006.1 using an air-oven. 

### 2.3. Analysis of Polyphenols and Organosulfur Compounds 

#### 2.3.1. Extraction of Free Compounds 

The extraction of polyphenolic and organosulfur compounds was carried out as reported previously by Inchikawa et al., [21], with few modifications. Briefly, 1 g of garlic clove powder or 0.5 g of garlic skin powder were homogenized with 5 mL of 80% methanol containing 0.01 N of HCl for 30 s at maximum speed (IKA Ultra-Turrax T-25 Digital Homogenizer, Staufen, Germany). The homogenate was subsequently placed in an ultra-sonic water bath at room temperature for 30 min to support the release of bioactive compounds, followed by centrifugation at 2500 rpm for 5 min at room temperature (Eppendorf Centrifuge 5804, Hamburg-Eppendorf, Germany). Supernatants were retained, whilst the residues were re-extracted with 80% methanol containing 0.01 N of HCl and applied to ultra-sonication for another 10 min and centrifuged, as described above. Finally, the supernatants were combined and filtered through 0.2 μm membrane filters (GHP Acrodisc, Pall, Cheltenham, Victoria, Australia) for chromatographic analysis using ultra-high performance liquid chromatography coupled with photodiode array detection or mass spectrometry (UHPLC-PDA or UHPLC-MS), oxygen radical absorbance capacity (ORAC), and total phenolic content (TPC) measurements. The extractions were conducted in triplicate. 

#### 2.3.2. Extraction of Bound Phenolic Compounds 

The extraction of bound phenolic compounds followed the method described by Adom and Liu [22] with modifications. Briefly, the residues obtained from the free phenolics extraction were subjected to alkaline hydrolysis by 2M of NaOH and shaken for 1 h at 200 rpm, using a reciprocating shaker (RP1812, Paton Scientific, Victor Harbor, South Australia, Australia). Then, the samples were acidified to pH 2.0 with concentrated HCl. Subsequently, ethyl acetate was added and mixed on a vortex for 30 s to extract the released bound-phenolic compounds into the organic solvent phase. The samples were centrifuged at 1500 rpm at room temperature for 5 min, and the upper phase was retained, while the lower phase was subjected to another three rounds of extraction with ethyl acetate, as described above. Supernatants were combined and dried under nitrogen at 40 °C in a dry block heater (DBH30D, Ratek Instruments Pty Ltd., Boronia, Victoria, Australia). The extracts were re-dissolved in 50% methanol containing 1% formic acid for further analysis. 

### 2.4. Total Phenolic Content and Antioxidant Capacity 

Total phenolic content (TPC) was measured by employing a Folin–Ciocalteu assay as reported previously [23], using a micro-plate absorbance reader (Sunrise, Tecan, Maennedorf, Switzerland) at 700 nm. TPC is expressed as milligrams of gallic acid equivalents per gram of sample (mg GAE/g), based on the standard curve obtained from the gallic acid at different concentrations (0 mg/L, 21 mg/L, 42 mg/L, 63 mg/L, 84 mg/L, and 105 mg/L). ORAC assay was performed followed the method developed previously [24], using a micro-plate reader (VICTOR3 2030 multilabel counter, PerkinElmer, Waltham, MA, USA) equipped with fluorescent filters (excitation at 485 nm and emission at 520 nm). Antioxidant capacity is presented as µMol of Trolox equivalents per gram of sample based on the standard curve obtained from the Trolox standard at different concentrations (0 µMol, 6.25 µMol, 12.5 µMol, 25 µMol, 50 µMol, and 100 µMol).

### 2.5. Quantification of Polyphenols and Organosulfur Compounds

#### 2.5.1. Phenolic Acids 

Phenolic acid extracts, including free and bound forms, were analyzed using a Waters Acquity^TM^ UPLC-PDA System (Waters, Rydalmere, New South Wales, Australia). The compounds were separated on a Waters HSS-T3 column (100 × 2.1 mm *i.d*; 1.8 μm) maintained at 40 °C, with 0.1% formic acid in Milli-Q-water (*v/v*) as eluent A and 0.1% formic acid in acetonitrile (*v/v*) as eluent B. The gradient program is as follows: 3 min, 5% B; 4.3 min, 20% B; 9 min, 45% B; 11 min, 100% B; 14 min, 100% B, and 17 min, 5% B. The flow rate was at 0.4 mL/min. Phenolic acids were quantified at 280 nm using the external calibration curves of phenolic acid standards, including p-hydroxybenzoic acid, vanillic acid, caffeic acid, p-coumaric acid, ferulic acid, and sinapic acid.

#### 2.5.2. Anthocyanins 

Anthocyanins in the garlic skin samples were analyzed using an Agilent 1290 Infinity UPLC-PDA System (Agilent Technologies, Santa Clara, CA, USA), following the methods of Gasperotti et al. [25] and Fredericks et al. [26]. A Waters C18 BEH column (100 × 2.1 mm *i.d*; 1.8 μm) maintained at 60 °C was used to separate the compounds, using 1% formic acid in Milli-Q water (eluent A) and 1% formic acid in acetonitrile (eluent B). The gradient program (time (min), % B) was (0.0, 8); (6.0, 15); (7.0, 90); (8.0, 90); (15.0, 8), with a flow rate of 0.45 mL/min. Anthocyanins were quantified at 520 nm, with an external calibration curve of cyanidin-3-glucoside (Cya-3-glc).

#### 2.5.3. Organosulfur Compounds 

Organosulfur compounds were analyzed and quantified according to the method developed by Ichikawa et al. [21], with modifications. A Waters BEH-Amide column (100 × 2.1 mm *i.d*; 1.7 μm) at 25 °C was used to separate the compounds, with 0.1% formic acid in Milli-Q water (eluent A) and 0.1% formic acid in acetonitrile (eluent B). 80% B was used isocratically, with a flow rate of 0.15 mL/min. The organosulfur compounds were quantified at 210 nm using an external calibration curve of L-alliin. 

#### 2.5.4. Identification of Polyphenols and Organosulfur Compounds

Peak identities of the detected phenolic acids, anthocyanins, and organosulfur compounds were confirmed using a Thermo high resolution Q Exactive mass spectrometer equipped with a Dionex Ultimate 3000 UHPLC system (Thermo Fisher Scientific Pty Ltd.). A full scan in both positive and negative (ESI) ionization mode was acquired at a resolving power of 70,000 full width half maximum. For the compounds of interest, a MS scan range of m/z 100–1200 was selected. Negative ionization mode was employed for all the phenolic acids, while positive ionization mode was applied for the identification of anthocyanins and organosulfur compounds. A data processing method using Thermo Xcalibur^TM^ software (Thermo Fisher Scientific Pty Ltd.) was employed to confirm the identities of individual compounds. 

### 2.6. Antimicrobial Screening Test

Freeze-dried garlic skin powder (1 g) and garlic clove powder (2 g) were extracted with hot water (80 °C) or methanol (60 °C) for eight cycles using a Dionex™ Accelerated Solvent Extraction (ASE) system (Dionex™, Sunnyvale, CA, USA). Following the extraction, the water and methanol extracts were evaporated at 60 °C and 40 °C, respectively in a centrifugal vacuum concentrator (miVac sample Duo concentrator) (Genevac Inc, New York, NY, USA) until dryness. Ethanol 20% (*v/v*) was used to reconstitute the extract precipitates prior to antimicrobial activity testing. 

Fresh microorganism colonies that had been revived from stock cultures for 24 h or 48 h (depending on growth) were dissolved into saline solution to reach the final absorbance reading of approximately 0.1 at 540 nm. The obtained bacterial solution was used to inoculate the standard agar plates. The disc diffusion method was applied for the antimicrobial activity test by placing a sterilized Whatman No. 1 Filter paper disc (13 mm *i.d*) onto the agar plates that had been inoculated with fresh bacterial solutions. One hundred μL of the reconstituted extract solutions from both the ASE water and methanolic extracts were added to the filter paper discs in triplicate. A negative control (ethanol 20%) was also included in the test. The agar plates were incubated at 37 °C for 24 h or 48 h (depending on growth), and the inhibition zones were recorded.

### 2.7. Statistical Analysis

A one-way analysis of variance (ANOVA), using Minitab 16 for Windows (Minitab Inc., State College, PA, USA), was applied to test the variances of measurements. A p value of 0.05 or less was used to determine significant differences. Chemometric data analysis was performed for data matrix, including six samples with triplicate values and 36 variables, using Unscrambler® X 10.3 (CAMO Software Inc., Magnolia, TX, USA). Data was normalized to similar weights for all the variables prior to principal component analysis (PCA). The PCA score plot and correlation loading plot were calculated for sample grouping and general evaluation of the correlation between the variables and sample characteristics.

## 3. Results and Discussion

### 3.1. Proximate

The results of the proximate analysis of garlic cloves show that all three samples have a relative similar composition, except for the protein content, which was higher in the Australian grown cultivars compared to the non-Australian grown garlic (22–23% DW versus 16.8% DW) (Table 1). In contrast, the total carbohydrate and sugar content in the Australian grown garlic were lower than in the commercial non-Australian grown garlic. The slight difference in the proximate composition probably reflects the differences in cultivars, growing conditions, and locations, as reported previously [27]. The minerals and heavy metals of all the samples were found to be in the range of the regulatory limits, as shown in Table 1. 

### 3.2. Total Phenolic Content (TPC)

**Figure 1:**
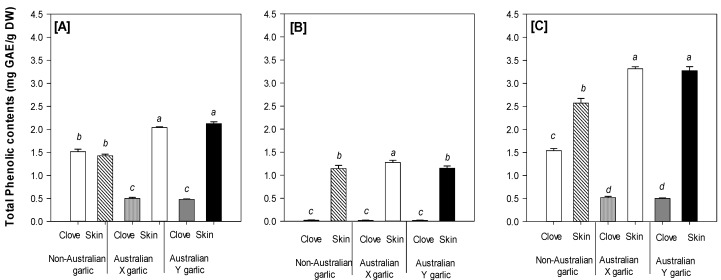
(**A**) Free, (**B**) bound, and (**C**) total (free + bound) TPC of different garlic cultivars and tissues. Data present mean ± SD (*n* = 3). Different letters in the same figure indicate significant difference at α = 0.05.

Figure 1 shows significant (*p* < 0.05) differences in the free, bound, and total TPC of the garlic samples studied. Overall, the free TPC was higher than the bound TPC; meanwhile, the free, bound, and total TPC were greater in the skin samples compared to the cloves, except for the free TPC in the commercial non-Australian grown garlic. Furthermore, the total TPC in the skin samples of the Australian grown garlic cultivars was significantly (*p* < 0.05) higher than in the commercial sample tested, whereas the cloves had a significant (*p* < 0.05) lower total TPC than the studied commercial garlic. The TPC results in the present study are in the same range as reported in the literature [15,27,32] and also in agreement with the findings of Nuutila et al. [33], who reported that the TPC in garlic skin is higher than in the cloves. Interestingly, the TPC of the Australian grown garlic was comparable with that of other garlic cultivars reported such as *Spanish Roja*, *Chinese Spring,* and *California White* [34]. 

### 3.3. Bioactive Compounds

#### 3.3.1. Phenolic Acids and Anthocyanins

Several phenolic acids and anthocyanins could be identified and quantified in the ‘free and bound’ extracts (Table 2 and Table 3) and were predominantly found in the garlic skin samples (Table 3). These findings support the obtained TPC results, which demonstrated a higher content of phenolic compounds in the skin compared to the cloves. The phenolic acid concentrations (free and bound) in the skin samples of the Australian grown garlic cultivars were significantly (*p* < 0.05) higher than in the commercial non-Australian grown garlic, whereas the cloves of the selected commercial sample contained more (*p* < 0.05) phenolic acids than the Australian grown cultivars. However, the amount of individual phenolic acids in the cloves of all the garlic samples was much lower than that in the garlic skin samples, as shown in Table 3. The main phenolic compounds found in the Australian grown garlic in the present study were slightly different to the polyphenolics of nine commercial garlic varieties grown in different countries [35]. This again reflects the impact of cultivars and environmental conditions on the polyphenolic composition in garlic.

Anthocyanins could only be detected in the skin samples of the Australian grown garlic with cyanidin-3-(6’-malonyl)-glucoside as the main anthocyanin in both cultivars (Table 3). The available information about the anthocyanins present in garlic is very limited. However, our findings are in agreement with previous studies [36,37], confirming that cyanidin-3-(6’-manolyl)-glucoside is the main anthocyanin in garlic leaves (skin).

#### 3.3.2. Organosulfur Compounds

**Figure 2:**
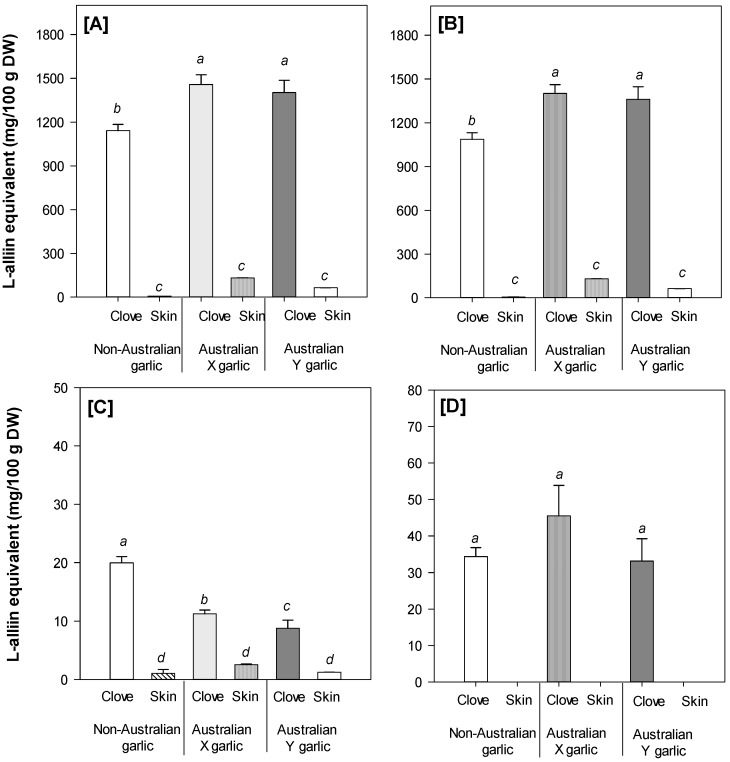
Total amount of organosulfur compounds (**A**) and individual organosulfur compounds, including L-alliin (**B**), alliin isomer (**C**), and methiin (**D**) in different garlic samples. Data present mean ± SD (*n* = 3). Different letters in the same figure indicate significant differences at α = 0.05.

Three different organosulfur compounds could be identified by UHPLC-PDA-MS, including L-alliin, an alliin isomer, and methiin (Table 4). L-alliin was the predominant compound contributing to more than 90% of the total amount of organosulfur compounds in the garlic cloves (Figure 2). This is in agreement with a previous study, identifying L-alliin as the main organosulfur compound in garlic bulb [2]. Overall, L-alliin was found at significantly (*p* < 0.05) higher levels in the garlic cloves than in the garlic skin samples. However, the concentrations of L-alliin and total amount of organosulfur compounds were significantly (*p* < 0.05) higher in the Australian grown garlic cultivars than that in the commercial non-Australian grown garlic (both cloves and skin; Figure 2). An isomer of L-alliin was also identified in the garlic samples, but at a very low concentration (Table 4 and Figure 2). This obtained result is in agreement with the findings reported by Ichikawa et al. [21]. Furthermore, the profile of organosulfur compounds in the garlic samples investigated in the present study was similar to that reported by Horníčková et al. [39], who investigated 58 different garlic genotypes, with L-alliin being found as the predominant compound, followed by an alliin isomer and methiin as the minor ones. The characteristic distribution of bioactive compounds between the garlic cloves and skin (more organosulfur compounds in the cloves, but more phenolic compounds in the skin) may also affect the bioactive properties of these various garlic tissues.

### 3.4. ORAC Assay

**Figure 3:**
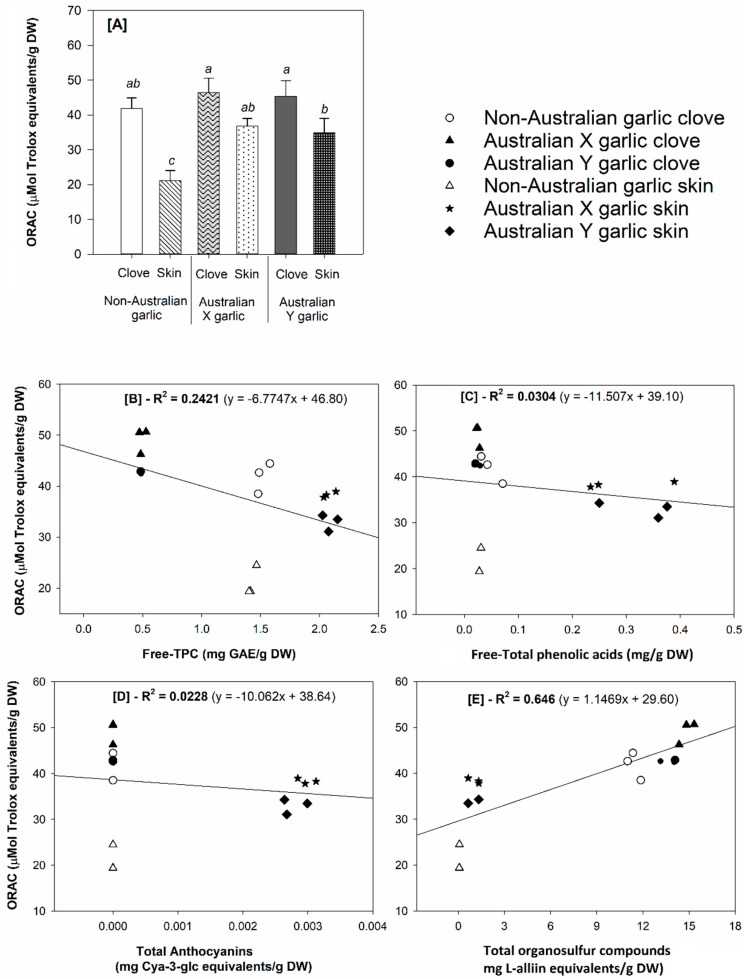
(A) Antioxidant capacity (Oxygen radical absorbance capacity—ORAC) and correlation between ORAC values and the determined (free) phytochemicals in the analyzed garlic samples: [B] ORAC vs. free total phenolic content (TPC), [C] ORAC vs. free total phenolic acids, [D] ORAC vs. total anthocyanins, and [E] ORAC vs. total organosulfur compounds. Different letters in Figure A indicate significant differences in antioxidant capacity among the samples tested at α = 0.05 (*n* = 3).

Figure 3 presents the ORAC results of the garlic clove and skin samples and shows a potential correlation between ORAC and the determined bioactive phytochemicals. The highest ORAC antioxidant capacity was found in the garlic clove samples (Figure 3). Particularly, the ORAC values of the Australian grown garlic cultivars were higher than those of the commercial non-Australian grown garlic (*p* < 0.05 for the skin samples and *p* > 0.05 (trend) for the cloves). In addition, the ORAC data had the strongest positive correlation with the organosulfur compounds (R^2^ = 0.646), which was considerably higher than the correlation with the TPC (R^2^ = 0.2421), phenolic acids (R^2^ = 0.0304), and anthocyanins (R^2^ = 0.0228) (Figure 3). This suggests that L-alliin is most likely responsible for the observed antioxidant capacity determined by ORAC. These results are in agreement with previous publications, which reported a strong correlation between antioxidant capacity and organosulfur compounds in a broad range of Allium vegetables, including garlic, onion, chive, shallot, Chinese leek, and hooker chive [42,43,44]. Furthermore, the superoxide and hydroxyl radical scavenging capacity of common organosulfur compounds, including L-alliin, allyl cysteine, allyl disulfide, and allicin have been previously demonstrated in the study by Chung [45]. 

### 3.5. Antimicrobial Activity

Garlic has been proved to be effective against a wide range of microorganisms [46]. The results of the antimicrobial activity testing of the methanolic and water extracts of the different garlic samples (cultivars and tissues) are presented in Table 5. Generally, there is variation in the antimicrobial activity between the Australian grown garlic and the commercial non-Australian grown sample and between different garlic tissues. The garlic clove samples clearly showed a stronger antimicrobial activity (extended inhibition zone) compared to the skin samples (Table 5), which is most likely due to the relatively high concentrations of organosulfur compounds (mainly L-alliin, which is a well-known strong antibacterial reagent) presenting in the garlic cloves. In addition, the results indicated a better antimicrobial effect induced by the methanolic extract of both the Australian grown garlic glove and skin samples (particularly cultivar X) compared to the studied commercial sample. While the skin samples of Australian grown garlic showed limited inhibitory effects to several bacteria and yeast, the commercial non-Australian grown garlic skin sample did not show inhibitory effects to any microorganism tested (Table 5). This suggests a promising application for the development of natural food preservatives from the extracts of the garlic cloves and garlic skin (potential utilization for the Australian grown garlic). 

The inhibitory effect is also found to be dependent on the type of solvent used for the extraction. For example, the methanolic extracts showed a greater inhibitory zone to most of the tested microorganisms (*p* < 0.05) compared to the water extracts of the corresponding samples (Table 5). The obtained results were not surprising, as methanol has been reported to be more efficient in the extraction of bioactive compounds in a garlic matrix compared to water [47,48]. The results of antimicrobial activity are in agreement with the results reported by the others that aqueous extracts of commercial freeze-dried garlic powder or fresh garlic cloves showed inhibitory effects to food-related bacteria, yeasts, fungi, and viruses. A strong inhibitory activity could be observed against *Candida albicans* and *Listeria monocytogenes*, but was less effective against *Escherichia coli* and *Staphylococcus aureus* [49,50]. These findings of antimicrobial activity warrant future studies determining/identifying the individual bioactive compounds that are responsible for the observed antimicrobial activity.

### 3.6. Multivariate Data Analysis

**Figure 4:**
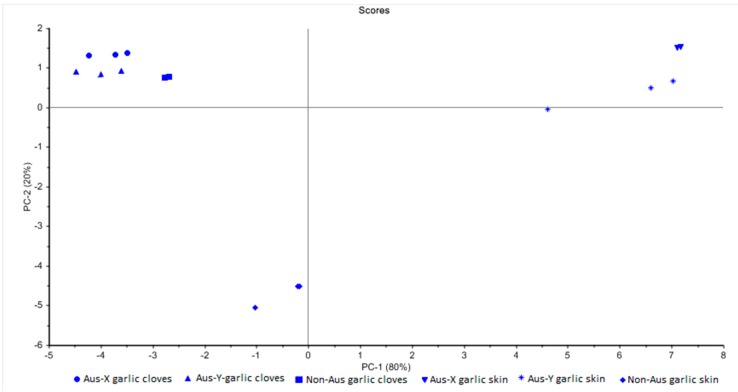
Principal component analysis (PCA) score plot classifies the samples into three distinguished groups (Aus: Australian).

The PCA score plot (Figure 4) classifies the samples studied in three distinguished groups: the clove samples of the three garlic cultivars, the skin samples of the Australian grown garlic, and the skin sample of the commercial non-Australian grown garlic. This finding supports the UHPLC-PDA-MS results, which could show significant (*p* < 0.05) differences in the phytochemical profiles between garlic skin and cloves as well as the Australian grown garlic and commercial non-Australian grown garlic. Furthermore, the anthocyanins that are only present in the skin samples of the Australian grown garlic also contributed to the differentiation between Australian grown garlic skin and studied commercial garlic skin (PCA score plot, Figure 4). 

**Figure 5:**
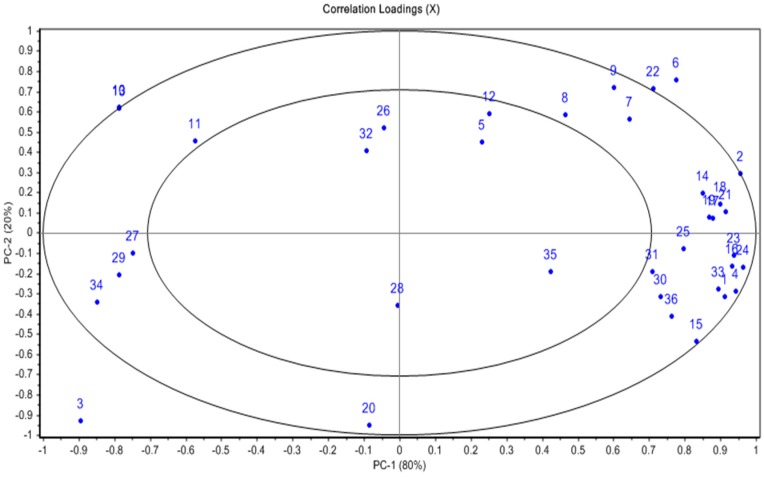
PCA loading plot describes all variables analyzed including polyphenols, organosulfur compounds, and associated bioactive properties.

The PCA correlation loading plot (PC1 versus PC2; Figure 5) helps correlate the samples and the variables measured. The PCA model predicts that the skin samples of the Australian grown garlic cultivars have a positive correlation with the determined phenolic compounds (both free and bound) as well as associated bioactive properties (ORAC and antimicrobial activity). In contrast, the skin sample of the commercial non-Australian grown garlic shows a negative correlation with almost all the measured variables, except for vanillic acid (Variable No. 3 and 20; Figure 5), indicating a lower total phytochemical content and subsequently limited exertion of bioactive properties. On the other hand, L-alliin, alliin-isomer, and the total organo-sulfur compounds correlate well with the clove samples of all three garlic cultivars studied. In addition, there is a positive correlation between the antimicrobial activity against a wide range of food-related microorganisms (e.g., *B. cereus* and *P. aeruginosa*; variables No. 27, 29, and 34) and the garlic clove samples (all the cultivars), predicting that the clove tissue and its bioactive phytochemicals are potential efficient antimicrobial ‘agents’.

Numbers listed in the PCA loading plot (Figure 5) are representing multiple variables as follows: (F: Free; B: bound, W: water extract; MeOH: Methanolic extract).

1. TPC-F10. L-Alliin19. Sinapic acid_B28. *L. monocytogenes*_W2. ORAC11. Allin isomer20. Vanillic acid_B29. *P. aeruginosa*_W3. Vanillic acid_F12. Methiin21. Total Phenolic acids_B30. *C. albicans*_W4. Caffeic acid_F13. Total organosulfur compounds22. Total Anthocyanins31. *R. mucilaginosa*_W5. p-Coumaric acid_F14. Total Phenolic acids_F23. *S. aureus*_W32. *B. cereus*_MeOH6. Ferulic acid- F15. TPC_B24. *E. coli*_W33. *L. monocytogenes*_MeOH7. Cyanidin-3-(6’-malonyl)-glucoside16. Caffeic acid_B25. *S. aureus*_MeOH34. *P. aeruginosa*_MeOH8. Cyanidin-based compound17. p-Coumaric acid_B26. *E. coli*_MEOH35. *C. albicans*_MeOH9. Pelargonidin-based compound18. Ferulic acid_B27. *B. cereus*_W36. *R. mucilaginosa*_MeOH

## 4. Conclusions

This study uncovered significant differences in the profiles of bioactive phytochemicals in different garlic cultivars as well as garlic tissues (skin and cloves). Both the skin and cloves of the Australian grown garlic cultivars were higher in bioactive phytochemicals than the commercial non-Australian grown garlic, which was an import from overseas. Furthermore, the garlic cloves could be identified as a rich source of organosulfur compounds (mainly L-alliin), resulting in a high antioxidant capacity and strong antimicrobial activity. Anthocyanins were only present in the skin of the Australian grown garlic, suggesting potential for the utilization as a by-product. However, detailed follow-up studies are warranted with more samples, quantity, and cultivars, in order to elucidate the potential of phytochemical-rich garlic for domestic and industrial applications (e.g., natural food preservatives), but also to further assess the nutritional value of garlic in a diverse and healthy diet.

## Figures and Tables

**Table 1:** Proximate analysis, minerals, and heavy metals of edible garlic cloves.

Proximate Composition		Unit	Non-AustralianGarlic Clove	AustralianX GarlicClove	AustralianY GarlicClove	DRI *
Energy		kJ/100 g	1454	1477	1457	
Protein		g/100 g	16.8	22.8	23.2	
Fat	Total fat	g/100 g	9.1	8.8	8.2	
	Saturated fat		2.0	1.9	1.8	
	Monounsaturated fat		0.4	0.6	0.7	
	Polyunsaturated fat		6.7	6.3	5.8	
	Trans fat		<0.01	<0.01	<0.01	
Carbohydrate	Total carbohydrate	g/100 g	31.6	28.4	28.3	
	Total sugar		3.2	3.0	2.7	
Dietary fiber	Total dietary fiber	g/100 g	36.9	34.9	34.7	
	Crude fiber		0.9	0.6	0.2	
Minerals	Sodium (Na)	mg/100 g	29	9.4	13	1.3 g AI [28]
	Potassium (K)		1,580	1,310	1,330	4.7 g AI [28]
	Iron (Fe)		2.9	2.4	1.7	8 mg RDA [28]
	Calcium (Ca)		47	34	36	1200 mg AI [28]
	Magnesium (Mg)		85	62	57	350 mg EAR [28]
	Zinc (Zn)		2.2	2.2	2.4	11 mg RDA [28]
Heavy metals	Mercury (Hg) Lead (Pb) Cadmium (Cd) Aluminum (Al) Chromium (Cr)	mg/kg	<0.01 <0.01 0.034 0.43 0.073	<0.01 <0.01 0.023 0.52 0.083	<0.01 <0.01 0.012 0.88 0.073	5 µg/kg BW/week UL [29] 25 µg/kg BW/week UL [29] 2.5 µg/kg BW/week UL [30] 1.0 mg/kg BW/week UL [31] 35 µg/day AI [29]
Moisture content		%	1.6	1.5	2.0	
Ash		%	3.9	3.5	3.5	

Data are based on dry weight [DW], * DRI—dietary reference intakes, RDA—recommended dietary allowance, AI—adequate Intake, UL—tolerable upper intake level, EAR—estimated average requirement; BW—body weight.

**Table 2:** Characteristics of phenolic acids and anthocyanins detected in the garlic samples. UHPLC: ultra-high performance liquid chromatography with photodiode array detection.

Tentative Identified Compound	Retention Time (min)	UHPLC-PDA *ʎmax* (nm)	[M–H]–m/z	Previous Reports
*Phenolic acids*				
Vanillic acid	3.6	280	167.0338	[14,27]
Caffeic acid	3.8	280	179.0438	[14,27,38]
p-Coumaric acid	4.6	280	163.0401	[13]
Ferulic acid	5.1	280	193.0495	[13,27,38]
Sinapic acid	5.3	280	223.0603	[13,14,27]
*Anthocyanins*			**[M–H]+ and fragment MS^2^**	
Cyanidin-3-(6’-malonyl)-glucoside)	3.5	520	535.1024, 287.0550	[17,36,37]
Cyanidin-based compound	5.2	520	862.2545, 538.1505, 287.0550	[16,37]
Pelargonidin-based compound	5.4	520	447.3906, 271.2058	

**Table 3:** Content of phenolic acids and anthocyanins in the garlic samples studied.

Phenolic Compounds	Non-Australian Garlic		Australian X Garlic		Australian Y Garlic	
	Clove	Skin	Clove	Skin	Clove	Skin
Free phenolic acids (mg/100 g DW)						
Vanillic acid	-	0.6 ± 0.06 a	-	0.4 ± 0.02 b	-	0.5 ± 0.02 b
Caffeic acid	0.1 ± 0.01 c (*)	0.6 ± 0.17 b	0.11 ± 0.04 c	0.9 ± 0.02 ab	0.13 ± 0.02 c	0.9 ± 0.02 a
p-Coumaric acid	-	-	-	8.0 ± 0.3 a	-	5.7 ± 0.4 b
Ferulic acid	-	1.7 ± 0.04 c	-	15.2 ± 0.8 b	-	30.5 ± 1.2 a
**Sum**	0.1 ± 0.01 d	2.9 ± 0.2 c	0.11 ± 0.04 d	24.4 ± 0.9 b	0.13 ± 0.02 d	37.5 ± 1.5 a
Bound phenolic acids (mg/100 g DW)						
Vanillic acid	0.02 ± 0.01 c	1.0 ± 0.06 a	-	0.2 ± 0.01 b	-	0.3 ± 0.03 b
Caffeic acid	0.04 ± 0.02 d	0.5 ± 0.04 c	0.02 ± 0.001 d	0.9 ± 0.04 b	0.02 ± 0.001 d	1.3 ± 0.05 a
p-Coumaric acid	0.05 ± 0.001 d	4.0 ± 0.28 c	0.2 ± 0.01 d	16.1 ± 0.3 b	0.14 ± 0.02 d	33.2 ± 0.5 a
Ferulic acid	0.07 ± 0.001 d	2.5 ± 0.05 c	0.1 ± 0.02 d	21.7 ± 0.6 b	0.11 ± 0.02 d	37.4 ± 0.5 a
Sinapic acid	0.4 ± 0.01 d	3.0 ± 0.34 c	-	6.9 ± 0.3 a	-	3.9 ± 0.2 b
**Sum**	0.6 ± 0.04 d	11.0 ± 0.7 c	0.3 ± 0.03 d	45.9 ± 0.6 b	0.4 ± 0.01 d	76.1 ± 1.1 a
Anthocyanins (mg Cya-3-glc equivalents/100 g DW)						
Cyanidin-3-(6’-malonyl)-glucoside	-	-	-	0.2 ± 0.02 a	-	0.2 ± 0.01 a
Cyanidin-based compound	-	-	-	0.03 ± 0.001 a	-	0.02 ± 0.001 a
Pelargonidin-based compound	-	-	-	0.1 ± 0.01 a	-	0.09 ± 0.01 a
**Sum**				0.3 ± 0.02 a		0.3 ± 0.02 a

(*) Data are mean ± SD (*n* = 3); different letters at the same row indicate significant differences at α = 0.05. (-): Not detected or presented in traces.

**Table 4:** Characteristics of organosulfur compounds detected in the garlic samples.

Tentative Identified Compound	Retention Time (min)	UPLC-PDA *ʎmax* (nm)	[M–H]–m/z	Previous Reports
L-alliin Alliin isomer Methiin	5.8 7.6 9.0	210 210 210	178.0532, 88.0398 178.0532, 88.0398 152.0375	[21,40,41] [21] [21,41]

**Table 5:** Antimicrobial activity of different garlic samples against food-related microorganisms (inhibition zone in mm).

Microorganism	Negative Control (20% Ethanol)	Non-Australian Garlic		Australian X Garlic		Australian Y Garlic	
		Clove	Skin	Clove	Skin	Clove	Skin
		Water extraction					
*B. cereus*	-	-	-	21.7 ± 1.2 a *	-	21.9 ± 1.3 a	-
*L. monocytogenes*	-	32.9 ± 0.5 a	-	33.9 ± 1.2 a	-	33.5 ± 1.5 a	-
*P. aeruginosa*	-	-	-	-	-	-	-
*C. albicans*	-	23.9 ± 0.7 a	-	23.5 ± 3.7 a	16 ± 0.5 b	24.5 ± 0.8 a	22.7 ± 1.3 a
*R. mucilaginosa*	-	27.2 ± 1.2 ab	-	27.1 ± 0.4 b	17.6 ± 2.4 d	30.7 ± 0.8 a	23.4 ± 0.8 c
*S.aureus*	-	18.9 ± 0.5 c	-	19.1 ± 1.1 c	22 ± 0.6 b	24.8 ± 0.6 a	-
*E. Coli*	-	16.9 ± 0.1 a	-	15.8 ± 0.7 a	16.8 ± 0.6 a	Possibly partial inhibition	-
		**Methanolic extraction**					
*B. cereus*	-	22.7 ± 0.6 c	-	30 ± 1.8 a	-	25.6 ± 1 b	13.3 ± 0.5 d
*L. monocytogenes*	-	24.9 ± 0.7 a	-	25 ± 1.5 a	19.2 ± 0.4 b	20.6 ± 0.8 b	19.2 ± 0.7 b
*P. aeruginosa*	-	-	-	13.5 ± 0.4 a	-	14.0 ± 0.5 a	-
*C. albicans*	-	38.3 ± 1.6 ab	-	40.9 ± 1.5 a	21.3 ± 1.1 c	37.1 ± 0.7 b	23.9 ± 1.2 c
*R. mucilaginosa*	-	37.3 ± 0.9 a	-	37 ± 0.4 a	24.0 ± 2.2 c	32.0 ± 1.1 b	27.3 ± 0.9 c
*S. aureus*	-	27.3 ± 4.7 ab	-	34.3 ± 2.1 a	20.5 ± 0.3 bc	30.0 ± 3.6 a	19.4 ± 0.5 c
*E. Coli*	-	19.8 ± 0.1 b	-	23.9 ± 0.6 a	-	19.6 ± 0.5 b	-

* Data expressed as mean ± SD (*n* = 3). Mean values of each row with different letters are significantly different (*p* < 0.05). (–) Denotes that no zone of inhibition was observed. Criteria for antimicrobial activity: <10 mm, weak; 10–15 mm, moderate and >15 mm, strong.
